# IL-1 and IL-23 Mediate Early IL-17A Production in Pulmonary Inflammation Leading to Late Fibrosis

**DOI:** 10.1371/journal.pone.0023185

**Published:** 2011-08-16

**Authors:** Paméla Gasse, Nicolas Riteau, Rachel Vacher, Marie-Laure Michel, Alain Fautrel, Franco di Padova, Lizette Fick, Sabine Charron, Vincent Lagente, Gérard Eberl, Marc Le Bert, Valérie F. J. Quesniaux, François Huaux, Maria Leite-de-Moraes, Bernhard Ryffel, Isabelle Couillin

**Affiliations:** 1 University of Orleans and CNRS, UMR6218, Orleans, France; 2 University of Paris V and CNRS, UMR8147, Paris, France; 3 INSERM U991, Université de Rennes 1, Rennes, France; 4 H2P2 Histopathological platform IFR140 INSERM U991, Université de Rennes 1, Rennes, France; 5 Novartis Pharma, Basel, Switzerland; 6 Institute of Infectious Disease and Molecular Medicine, University of Cape Town, Cape Town, South Africa; 7 Institute Pasteur, Laboratory of Lymphoid Tissue Development, CNRS URA 1961, Paris, France; 8 University Catholic of Louvain, Brussels, Belgium; 9 Key-Obs S. A S., Orleans, France; University of Pittsburgh, United States of America

## Abstract

**Background:**

Idiopathic pulmonary fibrosis is a devastating as yet untreatable disease. We demonstrated recently the predominant role of the NLRP3 inflammasome activation and IL-1β expression in the establishment of pulmonary inflammation and fibrosis in mice.

**Methods:**

The contribution of IL-23 or IL-17 in pulmonary inflammation and fibrosis was assessed using the bleomycin model in deficient mice.

**Results:**

We show that bleomycin or IL-1β-induced lung injury leads to increased expression of early IL-23p19, and IL-17A or IL-17F expression. Early IL-23p19 and IL-17A, but not IL-17F, and IL-17RA signaling are required for inflammatory response to BLM as shown with gene deficient mice or mice treated with neutralizing antibodies. Using FACS analysis, we show a very early IL-17A and IL-17F expression by RORγt^+^ γδ T cells and to a lesser extent by CD4αβ^+^ T cells, but not by iNKT cells, 24 hrs after BLM administration. Moreover, IL-23p19 and IL-17A expressions or IL-17RA signaling are necessary to pulmonary TGF-β1 production, collagen deposition and evolution to fibrosis.

**Conclusions:**

Our findings demonstrate the existence of an early IL-1β-IL-23-IL-17A axis leading to pulmonary inflammation and fibrosis and identify innate IL-23 and IL-17A as interesting drug targets for IL-1β driven lung pathology.

## Introduction

Idiopathic pulmonary fibrosis (IPF) represents a growing health issue, occurring from repeated lung injury of ill-defined origins. Patients suffering from this progressive form of interstitial lung disease present collagen accumulation and localised fibrotic foci in the absence of overt inflammation. Although chronic in nature, one complication is an accelerated phase of IPF. These exacerbations are characterized by acute inflammation reactivation which is frequently fatal within few weeks to a few months and underlining the important role of inflammation in IPF evolution [1]. Using the murine model of bleomycin (BLM)-induced lung fibrosis, we previously showed that inflammation and fibrosis were mediated through the pro-inflammatory and pro-fibrotic cytokine IL-1beta (IL-1β) and IL-1R1/MyD88 signaling in resident cells [2] [3]. Since interleukin-17 (IL-17) is a major pro-inflammatory cytokine involved in neutrophil recruitment [4], [5] and chronic lung pathologies [6], [7], we hypothesized that IL-1β secreted upon lung injury may induce innate “proTh17” IL-23 and IL-17 expression in lung resulting in pulmonary inflammation and evolution to fibrosis. Interestingly, a crucial role for IL-1 in the induction of IL-17-producing T cells that mediate autoimmune arthritis or EAE was reported, suggesting a link between IL-1 and IL-17 in establishment of some autoimmune diseases [8], [9]. IL-1β and IL-23 were shown to induce innate IL-17 production from γδ T cells amplifying Th17 responses and autoimmunity [10]. More recently, a study showed that bleomycin and IL-1β-mediated pulmonary fibrosis was IL-17A dependent [11]. IL-17 was first described to be produced by a CD4^+^αβ T cell subset Th17, characterized by IL-17 (or IL-17A), but also IL-17F, IL-21, IL-22, IL-6 and TNF-α production [12], [13]. The generation of CD4^+^ Th17 subset is under the control of specific cytokines and transcription factors [14]. In mice, TGF-β is necessary and the switch to the Th17 pathway is induced by IL-6 and/or IL-21 [15] and requires IL-23 for full *in vivo* differentiation, expansion and migration into the circulation and peripheral tissues [16], [17]. Similar to IL-17A, IL-17F signals via a receptor complex composed with IL-17RA and IL-17RC. The relative contribution of IL-17A and F in lung fibrosis was still unknown. IL-17A was also shown to be produced very early by γδ T cells triggering protective immunity against infection [18] exacerbating chronic inflammation [19]. During an immune response, γδ T cells rapidly produce IL-17 in response to IL-23 and/or other dendritic cell products while antigen-specific CD4^+^αβ^+^ Th17 cells develop later [20], [21]. Recently NKT cells that lack the NK1.1 marker were also identified as early IL-17-producing cells that can contribute to neutrophil recruitment through IL-17 secretion [22]. IL-17 synthesis depends on the RORγt transcription factor in both IL-17 producing CD4^+^αβ Th17 cells, and γδ T cells but also iNKT17 cells [23], [24], [25].

Here, we addressed the respective role of IL-17A and F, and the cellular source and activation cascade in response to BLM-induced airway injury. We show that lung injury triggers the expression of early IL-23, IL-17A and IL-17F in an IL-1 dependent manner, that early IL-23 and IL-17A, but not IL-17F are necessary for the establishment of the innate response to BLM and that γδ T cells are the major source of early IL-17A and IL-17F. Moreover, IL-23p19 is required for the late evolution to pulmonary fibrosis. Importantly, IL-23p19 and IL-17A are upstream of the expression of TGF-β1 the central mediator of lung fibrosis suggesting that innate IL-17A from γδ T cells [26] might promote the commitment towards an inflammatory Th17 phenotype through induction of TGF-β1 in an IL-6 rich environment.

## Methods

### Mice

IL-1R1−/− [27], IL-23/p19−/− [28] and IL-17RA−/− [29] backcrossed 10 times on the wild-type C57BL/6 genetic background were bred in our animal facility at the Transgenose Institute (CNRS, Orleans). *Rorc(*γ*t)-Gfp* ^TG^ were used for IL-17 producing cells investigation [30]. All animal experiments complied with the French Government's ethical and animal experiment regulations. This protocole was analysed and approved under the number CL2007-033 by the Ethic commitee for animal experimentation of CNRS and University in Orleans.

### Treatments

Bleomycin sulfate (5, 7.5 or 10 mg/kg) from Bellon Laboratories (Montrouge, France), rmIL-1β (50 µg/kg) from R&D Systems (Minneapolis, USA) were given intranasally in 40 µL under light ketamine-xylasine anesthesia. IL-17A (100 µg, Novartis Pharma, Basel, Switzerland), IL-17F (100–150 µg, Dr C. Uyttenhove, Bruxelles, Bergium) neutralizing antibodies or control Ig were administered intranasally or intraperitonally. A dose-response study was performed to find the effective dose for both antibodies.

### Bronchoalveolar Lavage Fluid (BALF)

BALF was performed as previously described [3].

### Lung homogenization and analysis

Lungs were homogenized and lung tissue myeloperoxidase activity (MPO) was evaluated as described [3].

### Cell count and determination

Total cell and differential cell counts were determined in BALF as previously described [3].

### Mediator measurements by ELISA

IL-1β, KC, IL-6 and TIMP-1 levels in BALF or lung homogenate were determined by ELISA (R&D system, Minneapolis, USA). Latent or active TGF-β1 levels were assayed in BALF and lung homogenates by ELISA (R&D, Minneapolis, USA). Latent or active TGFβ-1 was not detected in lung homogenates using this method.

### Semi-quantitative PCR

IL-17A, IL-17F, IL-23/p19 or IL-1β mRNA levels in lung were assessed (Online supplemented material). The electrophoresis gels were analyzed using a densitometric analyzer (ImageJ). Band densities were measured and compared to housekeeping gene HPRT1.

### Zymographic analysis of MMPs

MMP-2 activity was determined by gelatin zymography as described [2].

### Histology

After BAL and lung perfusion, the large lobe was fixed and 3-µm sections were stained with Hematoxylin and Eosine (H&E) or sirius red. For collagen determination the slides were stained with 0.1% picrosirius solution (0.1% sirius red in satured aqueous picric acid, pH 2) for 1 h, counterstained with Mayer's hematoxylin for 2 min. All the slides were numerised with Nikon i80 – X10 plan APO objective and QICAM color cooling camera (Qimaging). The lung sections were analyzed by imageJ - NIH software. The percentage of collagen was determined.

### Total lung collagen assay

Aliquots of lung homogenate (50 µl) were assayed for total lung collagen levels using the Sircol collagen dye binding assay according to the manufacturer's instructions (Biocolor Ltd, Northern Ireland).

### Flow cytometry analysis

Lung mononuclear cells were isolated and stimulated in the Online supplemented material. Cells were incubated with CD1d-tetramer-APC (NHI tetramer), anti-NK1.1-PerCP-Cy5.5, anti-CD4-APCalexa750, anti-CD8-PB, anti-TCRγδ-APC, anti-TCRαβ-APC, anti-CD11bPerCp Cy5.5 (BD PharMingen) or isotype control antibodies. For intracellular staining, cells were fixed with 4% PFA, washed, and permeabilized with 0.5% saponin (Sigma-Aldrich) in PBS, then incubated with anti-IL-17A-PE, anti-IL-17F-PE, anti-GFP alexa488, anti-IL12p40-FITC anti-IL-23p19-alexa647 or isotype controls. The cells were analyzed in a FACSCanto II (Becton Dickinson) by using FlowJo software.

### Statistical Analysis

Statistical evaluation of differences between the experimental groups was determined by non parametric one way analyse of variance test and Bonferroni post test using Prism software. *P* values of <0.05 were considered statistically significant.

## Results

### Early IL-23p19, IL-17A and IL-17F expression after airway bleomycin or IL-1β administration

**Figure 1:**
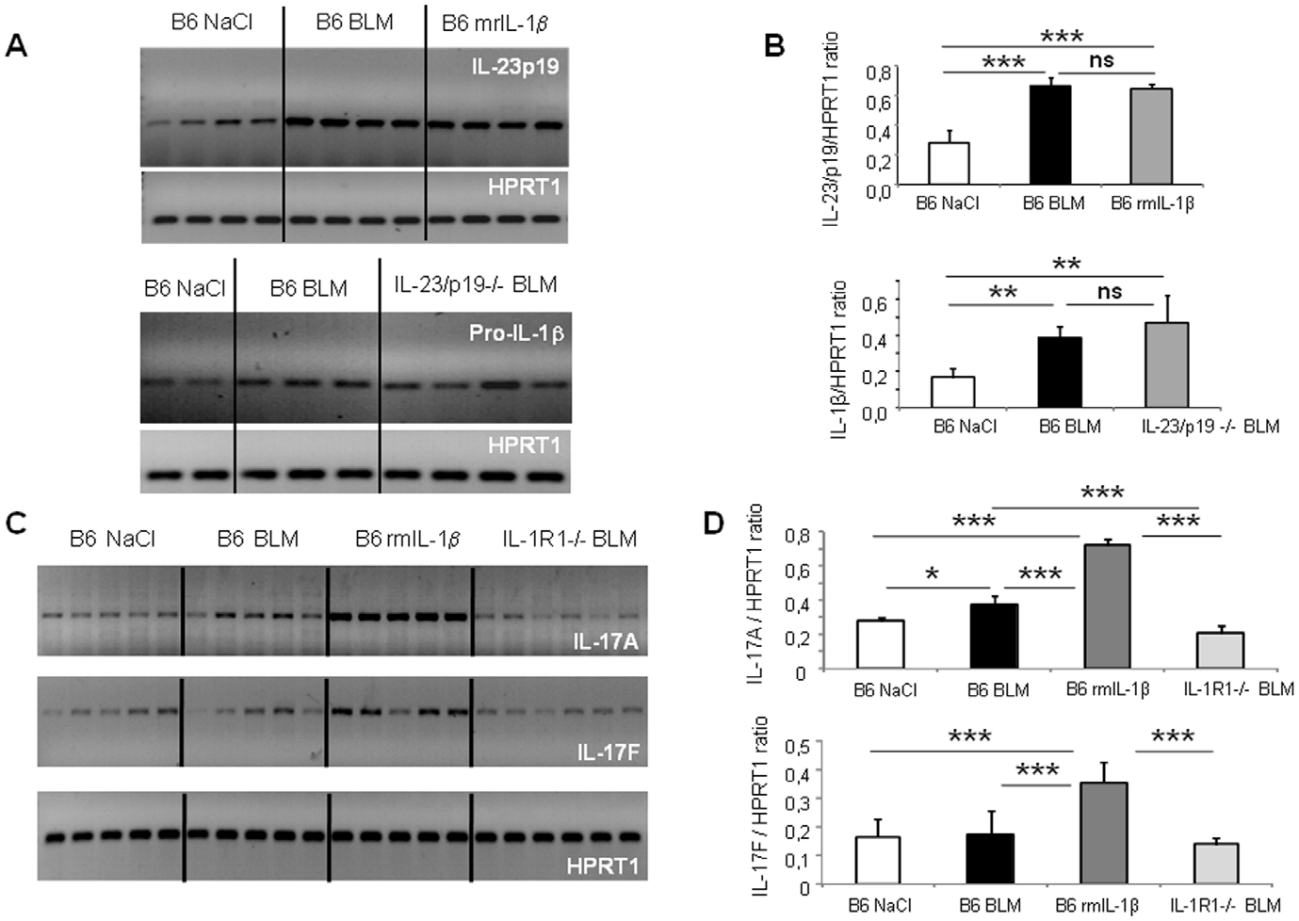
IL-23p19, IL-17A or IL-17F expression upon BLM or rmIL-1β. mRNA expression for IL-23p19, Pro-IL-1β and HPRT1 in lung of 4 individual mice treated with BLM (10 mg/kg) or rmIL-1β (50 µg/kg) or saline were assessed using semi-quantitative PCR at 24 h in wild-type mice (A). Semi-quantitative IL-1β expression in the lung, evaluated with IL-1β/HPRT1 PCR ratio, was reduced in absence of IL-23p19 signaling (B). mRNA expression for IL-17A, IL-17F and HPRT1 in lung of 5 individual mice treated with BLM (10 mg/kg) or rmIL-1β (50 µg/kg) or saline wild-type mice or IL-1R1−/− mice were assessed using semi-quantitative PCR at 24 h (C). IL-17A and IL-17F mRNA levels were compared to the housekeeping gene HPRT1 mRNA levels and expressed as IL-17/HPRT ratio by measuring band densities using a densitometric analyzer (ImageJ) (D). Data represent in FiguremRNA expression Data represent mean values ± SD from 3 ratio independent experiments (n = 4 or 5 mice per group; *, *p*<0.05; **, *p*<0.01; ***, *p*<0.001).

We previously showed that inflammation, remodeling and fibrosis after bleomycin (BLM) exposure were mediated through IL-1β release with recombinant murine IL-1β (rmIL-1β) recapitulating the effects of BLM [2]. We speculated that IL-1β secreted upon lung injury may induce innate IL-23 and IL-17 expression in lung resulting in pulmonary inflammation and evolution to fibrosis. Here we analyzed the expression of IL-23/p19 in the lung upon local BLM or rmIL-1β. Using semi-quantitative PCR analysis, we showed that IL-23/p19 mRNA expression is increased in lung of wild-type mice, 24 h after a single intranasal administration of BLM (Fig. 1A). Since IL-1β is induced following BLM, we asked whether rmIL-1β could also cause IL-23p19 expression. Indeed, instillation of rmIL-1β induced a significant increase of IL-23p19 pulmonary mRNA expression (Fig. 1A, B). Moreover BLM-induced pro-IL-1β expression in the lung was not affected in absence of IL-23p19 indicating that IL-1β is upstream of IL-23p19 (Fig. 1A, B). These results suggest that IL-1β produced locally upon lung injury induce IL-23p19 expression in the lung. Intracellular staining of IL-23p19^+^ cells by flow cytometry confirmed that IL-23p19 is produced by lung infiltrating CD11b^+^ monocytes (not shown). We then analyzed the respective expression of IL-17A and IL-17F in the lung after local BLM or rmIL-1β administration. We showed that IL-17A but not IL-17F mRNA expression is induced in the lung 24 h after BLM in these experimental conditions (Fig. 1C, D). Instillation of rmIL-1β induced a dramatic increase of IL-17A and IL-17F pulmonary mRNA expression (Fig. 1C, D). IL-17A and F expression after local BLM in IL-1R1 deficient mice was reduced in absence of IL-1R1 signaling suggesting that IL-1β produced locally upon lung injury may in turn induce IL-17A and IL-17F expression in the lung (Fig. 1C, D). These data show the existence of an IL-1β-IL-23-IL-17A axis following lung injury.

### Early pulmonary inflammation is dependent on IL-23p19 and IL-17RA signaling

**Figure 2:**
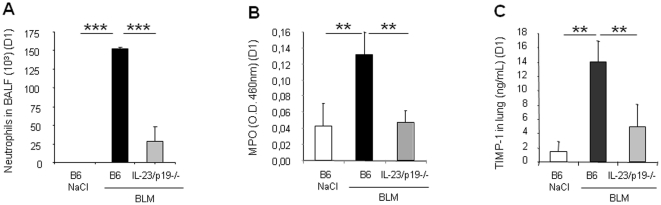
Acute inflammation and remodeling upon BLM are dependent on IL-23p19. Analysis of acute inflammation and remodeling was performed 24 h after BLM instillation (7.5 mg/kg). IL-23p19 deficient mice showed reduced neutrophil recruitment in BALF (A) and lung tissue (B) and reduced TIMP-1 production in lung (c) in comparison to wild-type mice Data represent mean values ± SD from 2 independent experiments (n = 5 mice per group; **, *p*<0.01; ***, *p*<0.001).

**Figure 3:**
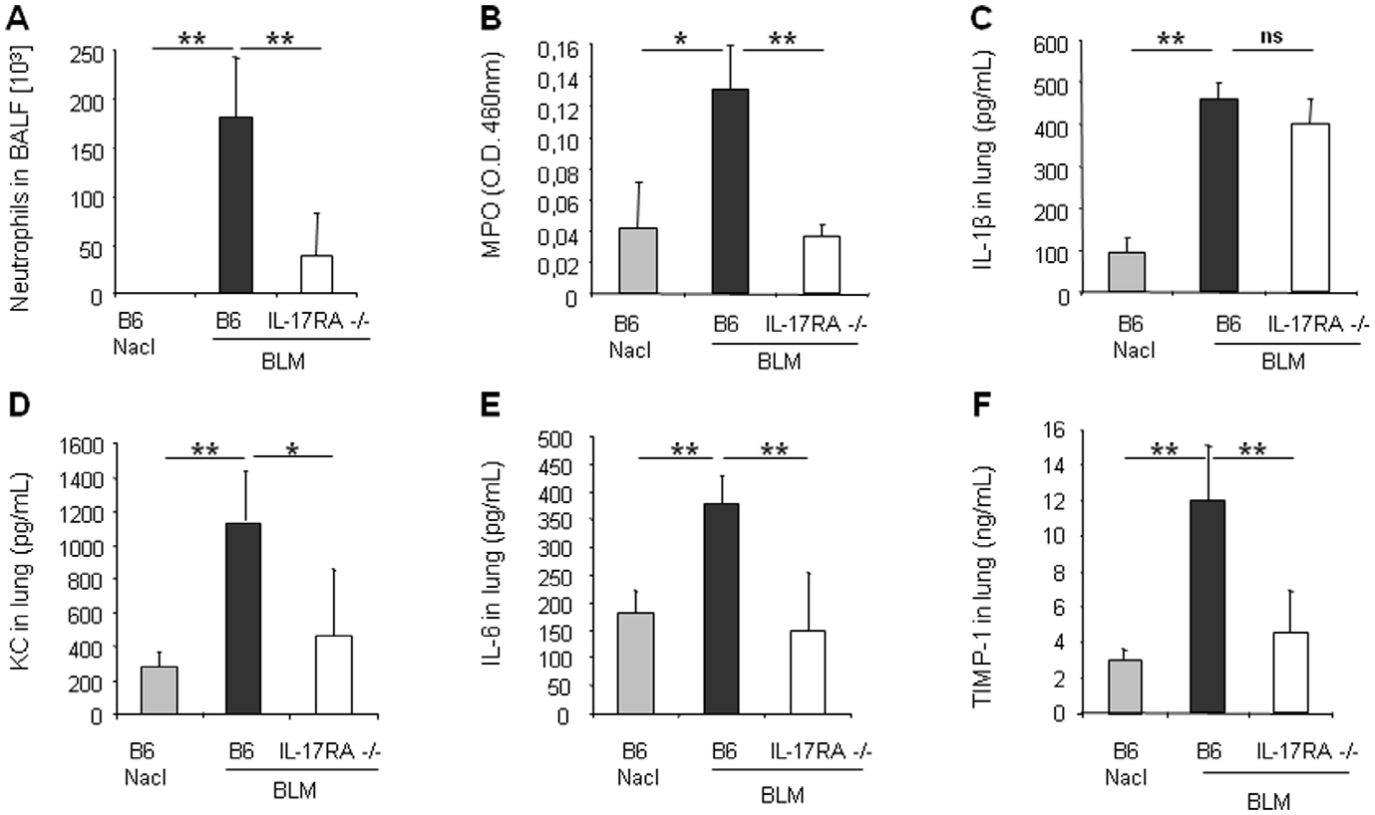
Impaired acute lung inflammation upon BLM in IL-17RA deficient mice. IL-17RA deficient mice showed reduced neutrophil recruitment in BALF (A) and lung tissue (B) in comparison to wild-type mice. BALs and myeloperoxidase (MPO) activity were performed 24 h after BLM instillation (7.5 mg/kg). KC (D), IL-6 (E) and TIMP-1 (F) levels in the lung were also reduced in IL-17RA deficient mice at 24 h but not IL-1β levels (C). Cytokines, chemokine and TIMP-1 quantification in lung homogenates were performed by ELISA. Data represent mean values ± SD from 4 independent experiments (n = 5 mice per group; ns, not significant, *, *p*<0.05; **, *p*<0.01).

To investigate whether IL-23p19 plays a role in lung inflammation upon lung injury, we evaluated early airway inflammatory cell response after airway BLM administration in lung of IL-23p19 deficient mice. Within 24 h, BLM elicited an acute neutrophil recruitment into the bronchoalveolar lavage fluid (BALF) and into the lung in C57BL/6 wild-type mice (Fig. 2A, B). In the absence of IL-23p19, neutrophil recruitment in the BALF (Fig. 2A) and neutrophil activity in the lung as assessed by MPO activity measurement (Fig. 2B) were dramatically reduced. Saline administration did not cause neutrophil influx in either group. IL-6 and KC production in lung 24 h after BLM were also diminished in the absence of IL-23p19 but IL-1β release was unaffected (not shown). To evaluate the role of IL-23p19 in the possible protease/antiprotease imbalance, we measured tissue inhibitor of matrix metalloproteinase-1 (TIMP-1) levels into the lung after BLM administration. Production of TIMP-1 is the hallmark of evolution towards fibrosis. Twenty four hours after BLM administration, lung TIMP-1 was significantly increased in wild-type mice but not in IL-23p19 deficient mice (Fig. 2C). Thus, BLM induced airway neutrophil recruitment, KC, IL-6 and TIMP-1 expression in an IL-23p19 dependent way, while IL-1β release was likely upstream of IL-23. To assess whether innate IL-17A and/or IL-17F play a role in early lung inflammation, we evaluated the inflammatory cell response in the lung of mice deficient for IL-17 receptor (IL-17RA). In the absence of IL-17RA, neutrophil recruitment in the BALF (Fig. 3A) and neutrophil activity in lung (Fig. 3B) were dramatically reduced 24 h post BLM. Production of both KC (Fig. 3D) and IL-6 (Fig. 3E) in the lung were reduced in IL-17RA deficient mice but IL-1β levels were not significantly modified, indicating that IL-1β is upstream of IL-17 (Fig. 3C). Moreover 24 h after BLM administration, lung TIMP-1 expression was induced in wild-type mice but not in IL-17RA deficient mice (Fig. 3F). Thus, BLM induced airway neutrophil recruitment, KC, IL-6 and TIMP-1 expression in an IL-17RA dependent way, while IL-1β release was likely upstream of IL-17.

### IL-17A, but not IL-17F, is essential for bleomycin-induced acute lung inflammation and remodeling

**Figure 4:**
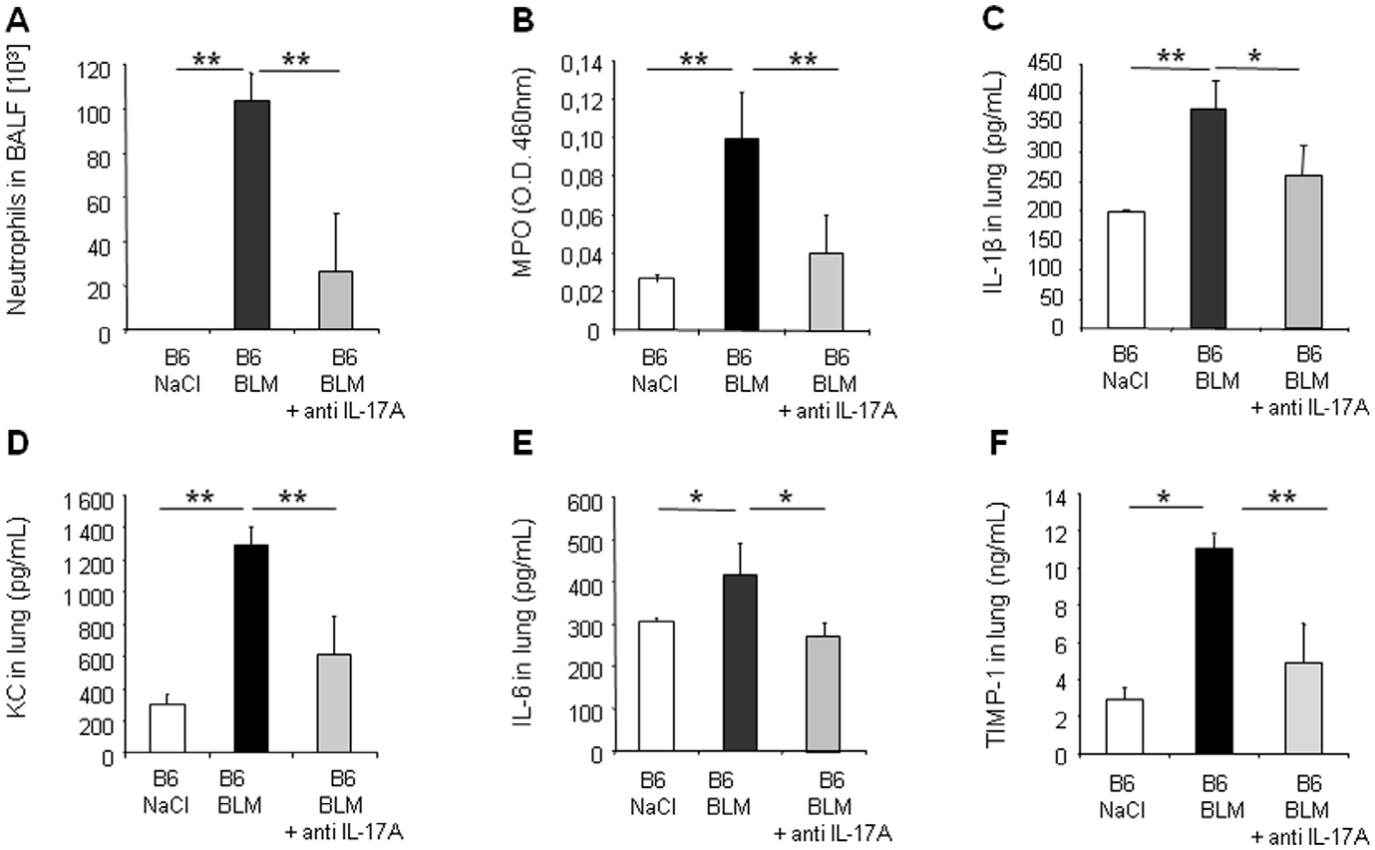
Acute lung inflammation and remodeling upon BLM depends on IL-17A. Inflammatory and remodeling responses of wild-type mice treated with neutralizing antibody against IL-17A (150 µg/mouse, i.p. just after BLM or saline i.n. instillation). Neutrophil recruitment in BALF (A) and myeloperoxidase (MPO) activity in lung tissue (B) induced by BLM (7.5 mg/kg) were reduced after mice treatment with anti-IL-17A. IL-1β (C), KC (D), IL-6 (E) and TIMP-1 (F) levels in lung homogenates assessed by ELISA were also reduced 24 h after mice treatment neutralizing antibody against IL-17A. Data represent mean values ± SD from 3 independent experiments (n = 4 mice per group; *, *p*<0.05; **, *p*<0.01).

**Figure 5:**
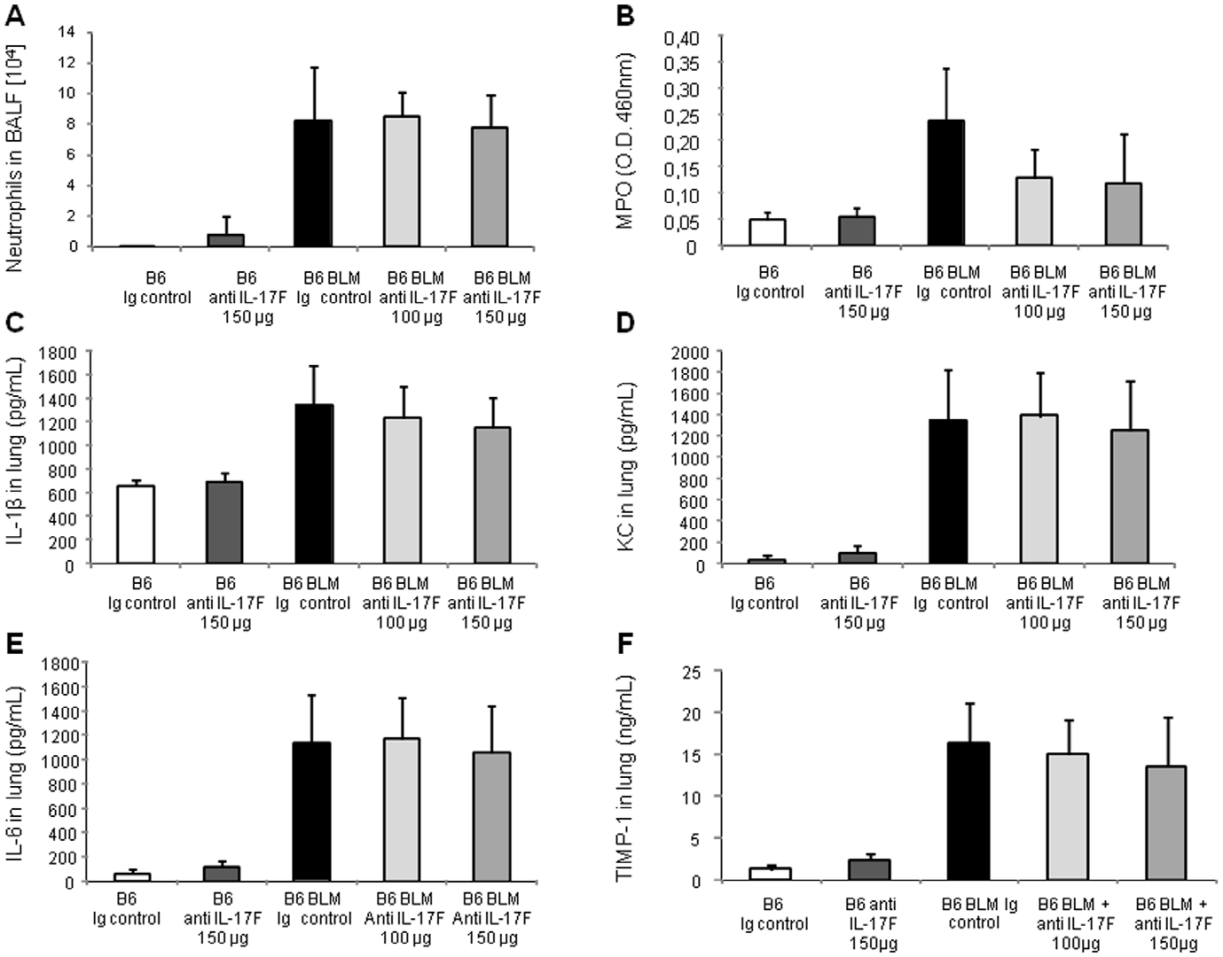
Acute lung inflammation and remodeling upon BLM are independent of IL-17F. Inflammatory and remodeling responses upon BLM (7.5 mg/kg) of wild-type mice treated with neutralizing antibody against IL-17F (100–150 µg/mouse, i.p. just after BLM or saline i.n. instillation). Neutrophil recruitment in BALF (A) and myeloperoxidase (MPO) activity (B) upon BLM (7.5 mg/kg) were not reduced 24 h after treatment with high doses of neutralizing antibody against IL-17F. IL-1β (C), KC (D), IL-6 (E) and TIMP-1 (F) in lung homogenates assessed by ELISA were also reduced in BLM mice 24 h after treatment with neutralizing antibody against IL-17F. Treatments with anti-IL-17F had not significant effect on all these parameters. Data represent mean values ± SD from 2 independent experiments (n = 6 mice per group).

To determine the relative contribution of IL-17A and IL-17F in BLM-induced acute lung inflammation, we investigated the inflammatory response to BLM using neutralizing antibodies against IL-17A or IL-17F. Neutralization of IL-17A led to a significant reduction of neutrophil recruitment into the BALF (Fig. 4A) and reduction of neutrophil MPO activity in the lung (Fig. 4B) 24 h after BLM administration. IL-1β (Fig. 4C), KC (Fig. 4D), IL-6 (Fig. 4E) or TIMP-1 (Fig. 4F) lung levels were also reduced after IL-17A neutralization. Lower IL-1β in mice treated with anti-IL17A could be explained by the existence of an autocrine or paracrine loop with IL-17 inducing IL-1β production and amplifying the response. In contrast, we could exclude a role of IL-17F in these responses by analyzing the effect of IL-17F neutralizing antibodies. Blocking IL-17F had no significant effect on neutrophil recruitment into the BALF (Fig. 5A) or into the lung parenchyma as shown by MPO analysis (Fig. 5B). Pro-inflammatory cytokines and chemokine levels were also not reduced by IL-17F neutralizing antibodies (Fig. 5C–E). Moreover, anti-IL-17F antibodies had no effect on lung TIMP-1 levels (Fig. 5F). Anti-IL-17A and anti-IL-17F antibodies were used at doses efficient in blocking lung neutrophilic inflammation induced by intranasal administration of rmIL-17A or rmIL-17F, respectively, to C57BL/6 wild-type mice (data not shown). Therefore, locally BLM-induced inflammation, but also repair processes depend on functional IL-17A, but not on IL-17.

### Cellular source of early IL-17A and F: γδ and αβ T cells but not iNKTcells

**Figure 6:**
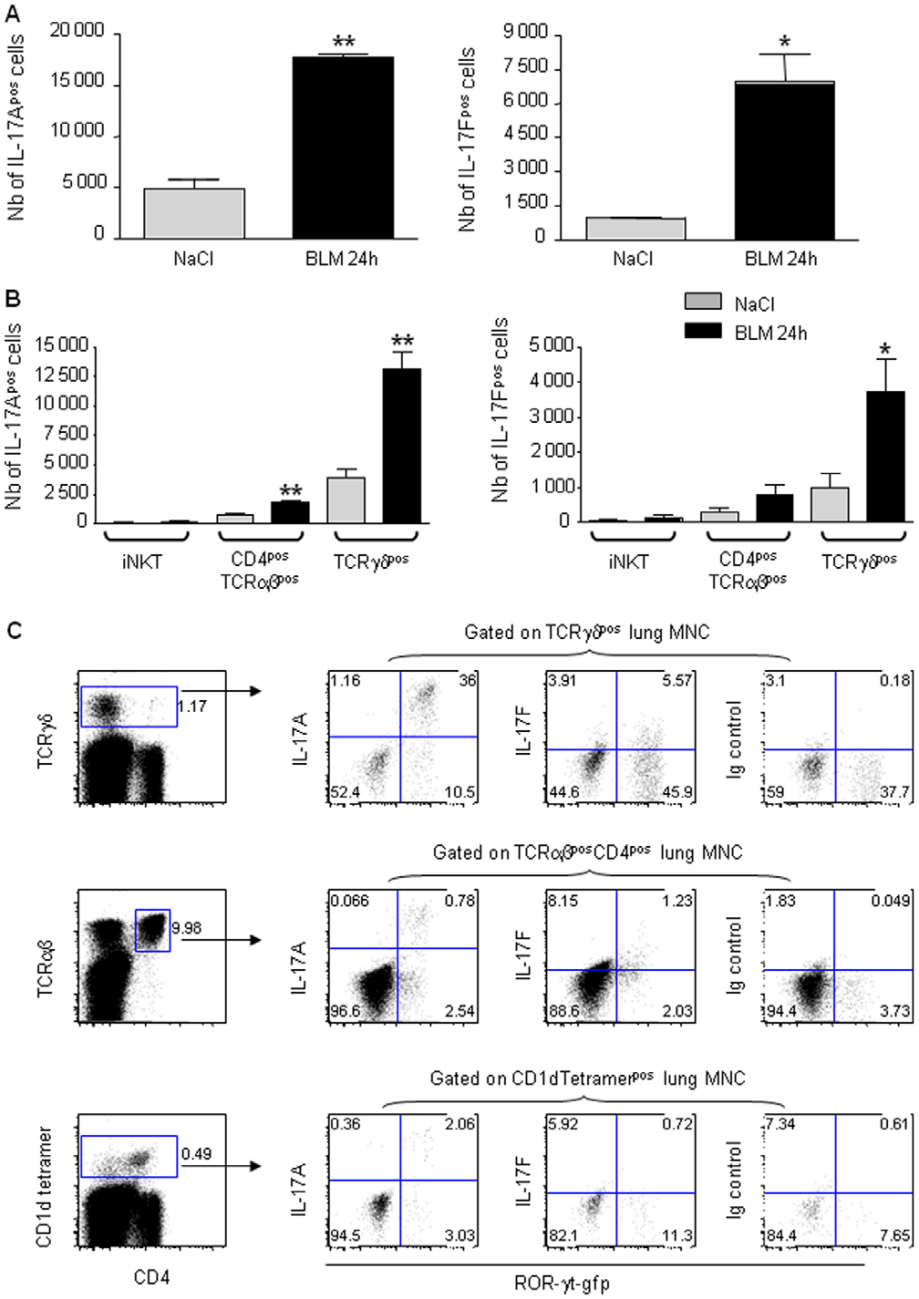
Early IL-17A and F are produced mainly by γδ T cells. Lymphocytes from the lung of *Rorc(γt)-Gfp* ^TG^ mice were prepared 24 h after BLM or saline administration, and stimulated in vitro with PMA/ionomycin for 4 h. Specific staining of cell markers (CD1d-tetramer-APC, anti-NK1.1-PerCP-Cy5.5, anti-CD4-APCalexa750, anti-CD8-PB, anti-TCRγδ-APC, anti-TCRαβ-APC), or isotype control and intracellular staining for IL-17 (anti-IL-17A-PE, anti-IL-17F-PE, anti-GFP alexa488 or isotype controls) were performed. FACS analysis showed that BLM administration enhanced the total number of IL-17A^pos^RORγt^pos^ and IL-17F^pos^RORγt^pos^ lung mononuclear cells (A). IL-17-producing cells positive for RORγt were essentially γδ T cells and CD4^pos^ αβ T cells but few iNKT cells (B). Among γδ T cells after bleomycin administration, at least 30% produce IL-17A and 5% produce IL-17F (C). Results are mean values ± SD from 4 independent experiments, with pools of 5 mice/group for each experiment, * p<0.05; **, *p*<0.01).

In order to investigate the IL-17 producing cells in the lung in response to bleomycin administration, we used transgenic *Rorc(γt)-Gfp* ^TG^ mice expressing EGFP under control of the *Rorc(γt)* promoter on a bacterial artificial chromosome (BAC) which allows visualization of the RORγt^+^ cells [24]. RORγt was shown to be a marker for IL-17–producing cells in both αβ and γδ T cell populations [24]. Lymphocytes from the lung of *Rorc(γt)-Gfp* ^TG^ mice were collected 24 h after BLM or saline administration, stimulated *in vitro* with PMA/ionomycin, and cell surface markers and intracellular staining for IL-17 were performed. FACS analysis showed that BLM administration enhanced the total number of IL-17A^pos^RORγt^pos^ and IL-17F^pos^RORγt^pos^ lung mononuclear cells (Fig. 6A). These IL-17-producing cells positive for RORγt were essentially γδ T cells and CD4^pos^αβ T cells but few iNKT cells (Fig. 6B). Up to 70% of IL-17A^pos^RORγt^pos^ cells and 60% of IL-17F^pos^RORγt^pos^ were γδ T cells. Among γδ T cells recruited after BLM administration, more than 30% produce IL-17A and 5% produce IL-17F (Fig. 6C). These data demonstrate that early IL-17A and F producing cells in response to BLM-induced lung injury are essentially γδ T cells and to a lesser extent CD4^pos^αβ T cells, while almost no iNKT cells produced IL-17A or F in these conditions.

### Lung remodeling and fibrosis are dependent on IL-23

**Figure 7:**
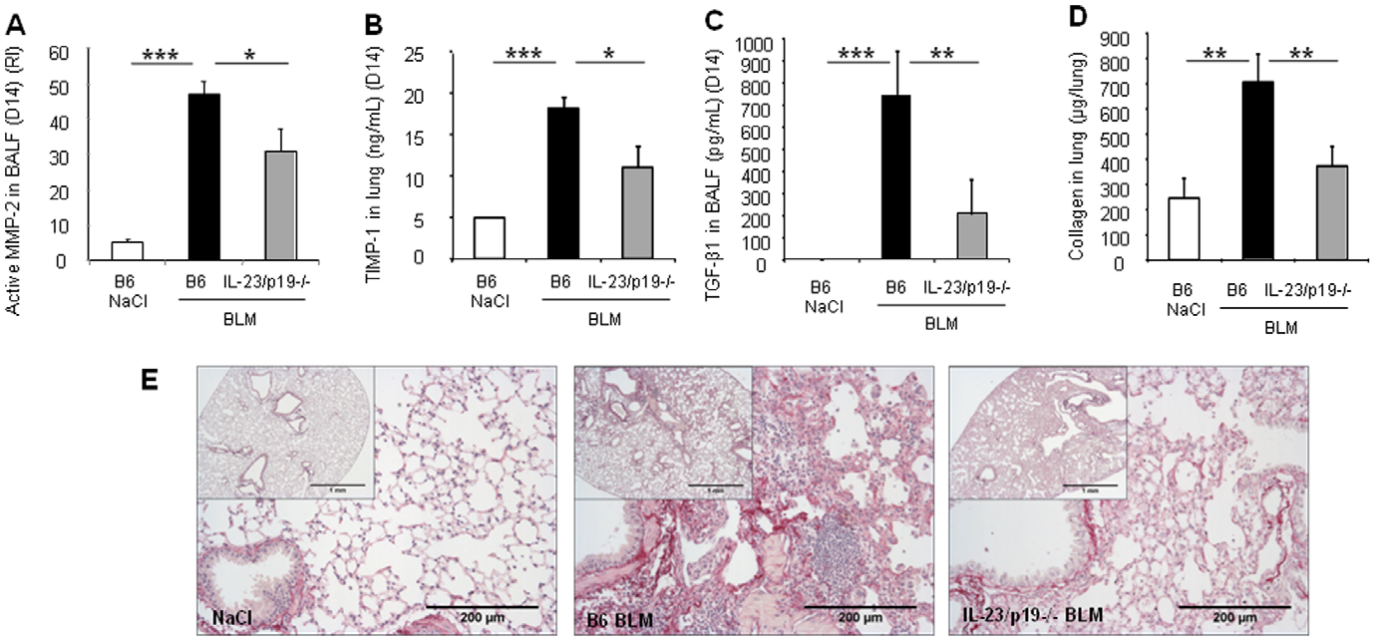
Remodeling and fibrosis are dependent on IL-23p19. Analysis of late pulmonary remodeling and fibrosis was performed 14 days after BLM administration (5 mg/kg). MMP-2 activity (A) and TIMP-1 production (B) were reduced in IL-23p19 deficient mice. Active MMP-2 was expressed as relative intensity (RI). Latent TGF-β1 detected in BALF from wild-type mice was reduced in BALF from IL-23p19 deficient mice (C). Total collagen content in lung was significantly reduced in IL-23p19 deficient mice in comparision to wild-type mice (D). Collagen was measured using the Sircol collagen dye binding assay. Lung microscopic sections showed extensive fibrotic areas with collagen deposition in wild-type mice treated with BLM which was attenuated in IL-23p19−/− mice (e). Sirius red (SR) staining, scale bars 1 mm and 200 µm, n = 5 mice. Data represent mean values ± SD from 2 independent experiments (n = 5 mice per group; *, *p*<0.05; **, *p*<0.01; ***, *p*<0.001).

To further assesss the role of IL-23 in lung fibrosis we analyzed remodeling and fibrosis in lungs of IL-23p19 deficient mice 14 days after BLM administration. MMP-2 (or gelatinase A, 72 kDa) is well described in pulmonary fibrosis, preferentially secreted by fibroblasts and epithelial cells [31]. Assessing gelatinase activity by zymography, we showed that BLM dramatically induced MMP-2 activity in wild-type mice and this activity was significantly reduced in IL-23p19 deficient mice (Fig. 7A). Moreover, BLM-induced TIMP-1 expression was also reduced in IL-23p19 deficient mice (Fig. 7B). Since TGF-β1 is essential in the development of lung fibrosis and also in the development of IL-17 producing cells in particular Th17 cells, we asked whether the production of TGF-β1 upon bleomycin is dependent on IL-23. Indeed, latent TGF-β1 was detected after activation in BALF from wild-type mice, 14 days after bleomycin administration but was reduced in BALF from IL-23p19 deficient (Fig. 7C). Active TGF-β1 was undetectable in BALF from wild-type and deficient mice probably due to lower production (data not shown). Microscopic analysis of lung sections showed extensive fibrotic areas with abundant collagen deposition as compared to normal alveolar structure of the saline controls 14 days post BLM. Cellular infiltrates, alveolar wall destruction and collagen deposition were significantly reduced in IL-17RA deficient mice (Fig. 7E) in comparison to BLM-treated wild-type mice. Collagen quantification in lung homogenates showed that total collagen content, enhanced 14 days after bleomycin instillation was significantly reduced in lung of IL-17RA deficient mice (Fig. 7D). These data demonstrate that lung remodeling and fibrosis require IL-23 for full responses *in vivo* and that IL-23 is involved in TGF-β1 production in response to epithelial lung injury induced by bleomycin.

### Bleomycin-induced remodeling, lung fibrosis and TGF-β1 are reduced in the absence of IL-17RA signaling and can be prevented by IL-17A neutralization

**Figure 8:**
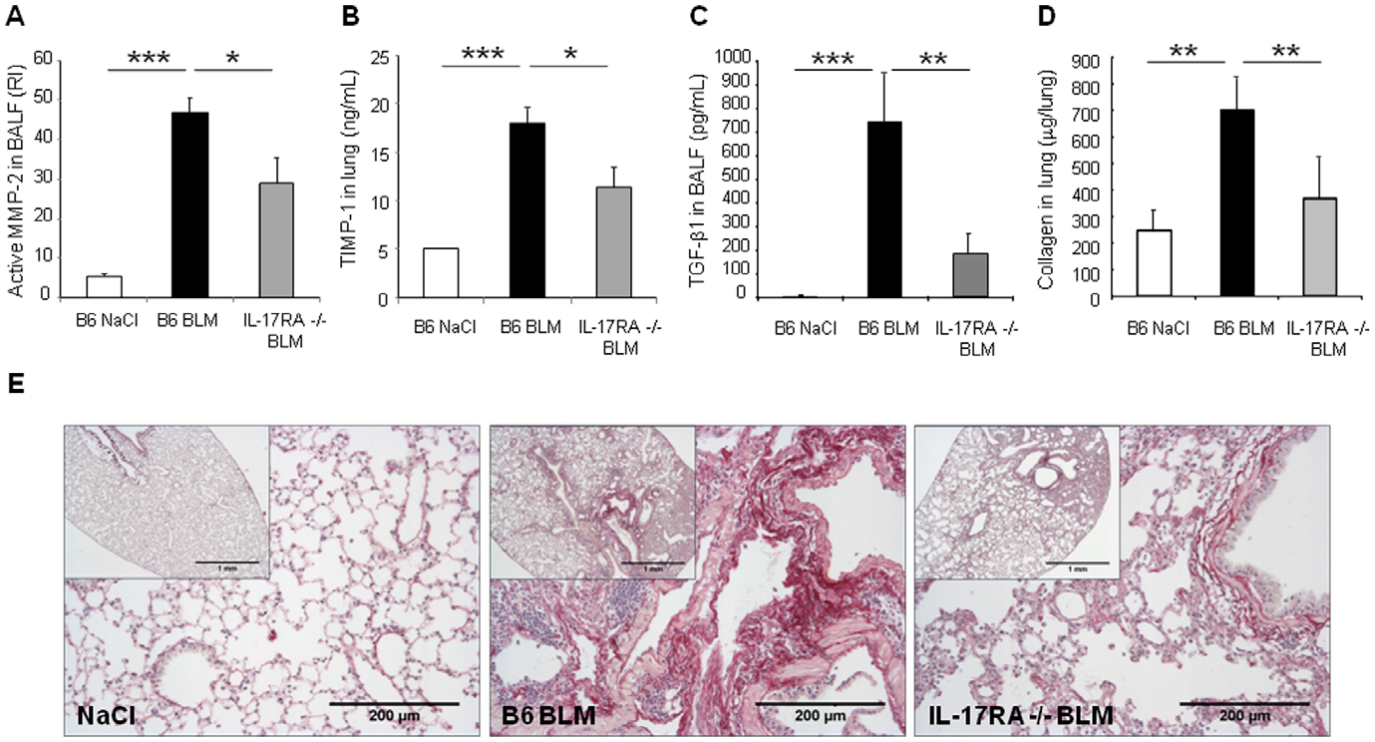
Attenuation of bleomycin-induced lung fibrosis in IL-17RA−/− mice. Pulmonary remodeling and fibrotic response of wild-type mice or IL-17RA deficient mice 14 days after BLM or saline i.n. instillation. MMP-2 (72 Kd) activity in BALF was analyzed by zymography 14 days after administration of BLM (5 mg/kg i.n.). Active MMP-2 was upregulated at day 14 after BLM in the BALF of wild-type mice but only partially in IL-17RA−/− mice (A). TIMP-1 as indicator of a fibrotic process was upregulated in the lungs of B6, but to a lesser extent in IL-17RA−/− mice on day 14 (B). The latent form of TGF-β1 was present in BALF from B6 mice, 14 days after BLM, but was reduced in BALF from IL-17RA deficient mice as determined by ELISA assay (C). Active MMP-2 was expressed as relative intensity (RI). TIMP-1 and TGF-β1 levels were assessed by ELISA. Total collagen content enhanced 14 days after bleomycin instillation was significantly reduced in lung of IL-17RA deficient mice (D). Collagen was measured using the Sircol collagen dye binding assay. Data represent mean values ± SD from 2 independent experiments (n = 4 mice per group; *, *p*<0.05; **, *p*<0.01; ***p<0.001). Lung microscopic sections showed extensive fibrotic areas at day 14 with collagen deposition in wild-type mice treated with BLM (5 mg/kg i.n.) which was attenuated in IL-17RA−/− mice (E). Sirius red (SR) staining, scale bars 1 mm and 200 µm, n = 5 mice).

Furthermore, the analysis of remodeling in lung of IL-17RA deficient mice 14 days after BLM administration showed that MMP-2 activity and TIMP-1 level were reduced (Fig. 8A, B). As latent TGF-β1 was reduced in BALF from IL-23-p19 deficient mice, we asked whether it required functional IL-17A. Latent TGF-β1 expression was also reduced in IL-17RA deficient mice (Fig. 8C) whereas active TGF-β1 remained undetectable (data not shown). Total collagen content in lung was significantly reduced in IL-17RA deficient mice in comparision to wild-type mice (Fig. 8D). Moreover, we observed that cellular infiltrates, alveolar wall destruction and collagen deposition were significantly reduced in IL-17RA deficient (Fig. 8E) in comparison to BLM-treated wild-type mice.

**Figure 9:**
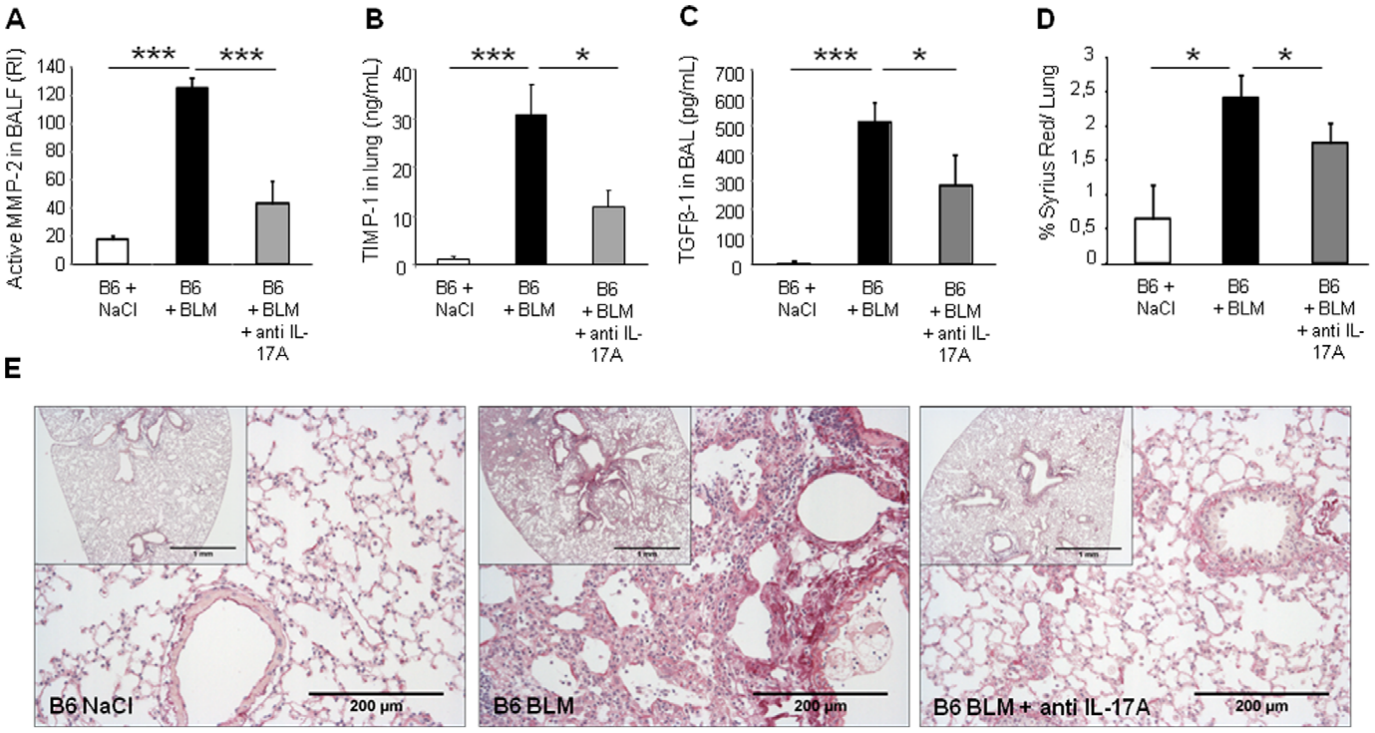
Attenuation of bleomycin-induced lung fibrosis upon anti-IL-17A treatment. Pulmonary remodeling and fibrotic responses of wild-type mice treated with neutralizing antibody against IL-17A (150 µg/mouse, i.p. just after BLM and 3 times per week) 14 days after BLM (5 mg/kg i.n.) or saline. MMP-2 (72 Kd) activity in BALF was analyzed by zymography 14 days after administration of BLM. Active MMP-2 upregulated upon BLM in the BALF of wild-type mice was greatly reduced after 6 treatments with neutralizing antibody against IL-17A (A). TIMP-1 in lung was also significantly decreased after treatment with neutralizing antibody against IL-17A in comparison to untreated BLM mice (B). Latent TGF-β1 detected in BALF from wild-type mice was reduced in BALF from wild-type mice treated with anti-IL-17A antibodies (C). Active MMP-2 was expressed as relative intensity (RI). TIMP-1 and TGF-β1 levels were assessed by ELISA. In order to quantify lung collagen deposition, all the slides were numerised by Qimaging and analyzed by imageJ - NIH software. % of SR by lung were shown. Collagen content enhanced after BLM was significantly reduced when the mice were treated with anti-IL-17A antibody (D). Data represent mean values ± SD from 2 independent experiments (n = 5 mice per group; *, *p*<0.05; ***, *p*<0.001). Lung microscopic sections showed extensive fibrotic areas at day 14 with collagen deposition in wild-type mice administrated with BLM which was attenuated in mice treated with neutralizing antibodies against IL-17A (E). Sirius red (SR) staining, scale bars 1 mm and 200 µm, n = 5 mice.

We further confirmed the implication of IL-17A in lung remodeling, and demonstrate that these developments can be prevented by anti-IL-17A neutralizing antibodies. Antibodies to IL-17A administered after BLM reduced MMP-2 activity in BALF (Fig. 9A), TIMP-1 level in lung (Fig. 9B) and latent TGF-β1 level in BALF (Fig. 9C) in wild-type mice. Moreover, anti-IL-17A antibody treatment attenuated pulmonary fibrosis observed at day 14 (Fig. 9E). Remodeling response, analyzed by collagen specific sirius red staining of microscopic lung sections showed that lung collagen content, enhanced after BLM, was significantly reduced by anti-IL-17A antibody treatment (Fig. 9D). Therefore, IL-17A and IL-17RA signaling are required for the development of pulmonary inflammation, fibrosis, and collagen deposition in response to bleomycin and IL-17A neutralization can prevent evolution towards fibrosis.

## Discussion

In this study, we report the involvement of IL-23p19 and innate IL-17A in the occurrence of pulmonary inflammation and fibrosis and demonstrate the critical role of IL-1β in these processes. We show the existence of an IL-1β/IL-23/IL-17 axis which is essential for bleomycin-induced pulmonary inflammation, remodeling and fibrosis. While this study was being finalized, Wilson and colleagues published a study reporting a critical role for IL-17A in BLM- or IL-1β-induced lung fibrosis [11].

IL-23p19 and IL-17A were expressed early in tissue following local bleomycin suggesting that these cytokines play a role in lung injury-induced inflammation and subsequent evolution to fibrosis. Wilson et al showed a weak IL-17A protein expression after restimulation of BAL and lung cells at day 4 and day 7 post BLM administration, respectively followed by a high IL-17A expression on day 7, 14 and 21 after bleomycin [11]. We previously outlined that exogenous IL-1β can mimic the BLM-induced pathology [2] and this was confirmed by Wilson et al [11]. We analyzed the effect of airway rmIL-1β on pulmonary IL-23p19 and IL-17 expression. IL-23p19, IL-17A and IL-17F mRNA were strongly increased after airway rmIL-1β administration and bleomycin-induced IL-17A and IL-17F expression were dependent on IL-1R1 signaling, demonstrating that IL-1β is upstream of IL-17. Further, IL-17 may promote IL-1β production by an amplification loop. Therefore the data suggest that lung injury promotes IL-1β production which increases IL-23 expression and in turn can stimulate innate IL-17 expression. An IL-1β/IL-17 axis has been reported in experimental autoimmune encephalomyelitis and arthritis for which a crucial role for IL-1β in the induction of IL-17-producing T cells was demonstrated [9], [10], [32]. Moreover IL-1β drives pathogenic Th17 cells during spontaneous arthritis in interleukin-1 receptor antagonist (IL-1Ra)-deficient [33]. Chronic destructive arthritis was reduced in IL-17RA deficient mice whereas abolished in IL-1R1 deficient mice [34]. In multiple sclerosis, IL-17 was produced by microglia in response to IL-23 or IL-1β [35]. In mice, IL-1β and IL-23 are produced by dendritic cells upon infection-induced Th-17 cells [36]. After lung injury, IL-1β may be produced very early by dendritic cells and alveolar macrophages acting on IL-17 producing cells to promote IL-17 expression. Our study showed for the first time that early IL-23p19 and IL-17RA signaling were necessary to trigger pulmonary inflammation and remodeling responses in response to lung injury. We confirmed the role of IL-17A in these responses [11]. Furthermore, we document the relative role of IL-17A versus IL-17F in lung inflammation leading to fibrosis, an aspect not known before [37]. We showed that IL-17A promotes pulmonary inflammation and remodeling upon airway BLM whereas IL-17F has no effect. IL-17A and IL-17F which are highly homologous members of the IL-17 protein family and bind the same receptor complex consisting of IL-17RA and IL-17RC were often described as pro-inflammatory cytokines with redundant role in inflammation and autoimmunity. IL-17F apparently plays a marginal role in the development of delayed-type and contact hypersensitivities, autoimmune encephalomyelitis, collagen-induced arthritis, and arthritis in IL-1Ra deficient mice. In asthma, IL-17F may play an important regulatory role and seems to function differently than IL-17A [37], [38]. Our results suggest that unlike IL-17A, IL-17F has no essential role in acute inflammation in our model. Overlapping or distinct roles of these cytokines may depend on IL-17A and/or IL-17F production by different cell types or on the role of functional homodimers or heterodimers. Since the role of innate IL-17 is less known and in an attempt to identify the cells producing IL-17 very early, we found γδ T cells but neither iNKT cells nor αβ T cells as the major source of innate IL-17A and IL-17F, 24 hrs after induction of lung injury. IL-17-producing γδ T cell were present in naïve mice and enhanced after bleomycin. Indeed, IL-17- producing γδ T cells may be differentiated in the periphery and poised ready to produce IL-17 in a rapid manner [39]. This is the first demonstration of early IL-17A and F production by γδ T cell after pulmonary injury. IL-17A producing γδ T cells are more present than IL-17F producing γδ T cells in agreement with the predominant role of IL-17A in pulmonary inflammation after injury. Late pulmonary induction of IL-17 expression by γδ T cells 7 and 14 days after local bleomycin was reported but the direct role of the IL-17 on late inflammation and fibrosis was not studied [6]. Moreover, in this study deletion of all γδ T cell was associated with reduced inflammation in the bronchoalveolar space but severe interstitial inflammation with complete loss of alveolar structure and delayed epithelium repair 7 and 14 days after bleomycin. By contrast, using mice deficient for IL-17 signaling or treated with anti-IL-17 antibodies, we show reduced inflammation in the bronchoalveolar space and in interstitium inflammation and confirmed two studies reporting that IL-17 has a detrimental role in lung fibrosis [6], [11]. Dendritic epidermal γδ T cells involved in skin epithelium wound repair [40] may also contribute to lung epithelium repair as suggested in the lung of tobacco smokers without COPD [41]. Depletion of these cells might be responsible for the disease aggravation described in γδ deficient mice [6]. Dendritic epidermal γδ T cells with lung wound repair activity and IL-17 producing γδ T cells with detrimental effect may coexist in the airways after lung injury. This could explain the different phenotypes observed between our study with mice deficient for IL-17 signaling and Braun and collagues's study with mice deficient for γδ T cells. As we show that early IL-17 producing cells are essentially γδ T cells, this suggests that IL-17 producing γδ T cells have a detrimental effect probably in inducing the expansion of Th17 which are auto-reactive and pathogenic T cells. γδ T cells were described as non-conventional T-cell implicated in early innate immunity whereas conventional CD4 αβ Th17 cells were often involved in adaptive immunity suggesting that late IL-17 producing cells in response to bleomycin-induced lung damages would be CD4 αβ Th17 cells [21]. In support of this, it was shown that IL-17-producing γδ T cells activated by IL-1β and IL-23 can promote IL-17 production by CD4^+^ T cells suggesting that γδ T cells act in an amplification loop for IL-17 production by Th17 cells [10]. Even if IL-17 production by γδ T cells has a protective role in inducing adaptive immune response against infection, IL-17 producing γδ T cells were implicated in inflammatory diseases associated with tissue damage [42], [43] and in the generation and activation of IL-17-producing auto-reactive T cells. IL-17-producing γδ T cells exacerbate collagen-induced arthritis and experimental autoimmune encephalomyelitis (EAE) [10].

Interestingly we report that airway TGF-β1, the main critical mediator of remodeling and fibrotic responses in the lung, was induced after lung injury in wild-type mice but strongly reduced in IL-23p19 and IL-17RA deficient mice or wild-type mice treated with anti-IL-17A antibodies indicating that IL-23p19 and IL-17A are upstream of the expression of TGF-β1 and suggesting that innate IL-17A produced by γδ T lymphocytes may influence the production of TGF-β1. In case of repeated injury, the emergence of TGF-β1 as a component associated with cellular injury and inflammation with IL-6 and IL-1β would provide the conditions for differentiation of CD4 αβ Th17 cells as a second wave of immune response. Wilson et al reported that IL-17A-induced fibrosis depend on TGF-β1. This may be explained by the existence of an amplification loop, early IL-17A inducing TGF-β1 and TGF-β1-dependent production of late IL-17A. Moreover, we showed for the first time that IL-23 is required for full establishment of pulmonary inflammation, remodeling and fibrosis in mice. IL-12p40 subunit, which associates with IL-23p19 subunit to form IL-23 cytokine, but neither IL-12p35 nor IL-12p70, was shown to play a key role in silica-induced pulmonary inflammation and fibrosis [44]. Increased serum IL-23 was observed in patients with systemic sclerosis [45]. Our results strongly support the notion that IL-23 is required for IL-17 production by γδ T cells in response to epithelial cell damage. Naïve γδ T cells have been shown to produce IL-17 in response to IL-23 alone [18], [39] and to constitutively express IL-23 receptor [10], [46]. These cells have a pathogenic role in severe disease such as collagen-induced arthritis [19]. In this case they appear to be driven by self molecules that arise during inflammation. Such a break of self tolerance with induction of pathogenic IL-17 producing γδ T cells should be possible in the case of repeated lung injury and inflammation leading to pulmonary fibrosis. Our study showed for the first time that IL-23p19 and IL-17RA signaling were necessary to trigger late pulmonary inflammation and fibrosis and confirmed by IL-17A neutralization that IL-17A is required for late disease [11]. Wilson's study and our work are largely in agreement or complementary although minor discrepancies may be due to the use of two different sources of bleomycin [11]. Another study described how IL-17A may regulate the expression and/or proinflammatory properties of IL-22 in pulmonary fibrosis but did bot analysed the link between IL-1β, IL-23and IL-17 [47].

In conclusion, we highlighted the existence of an innate IL-1β-IL-23-IL-17A axis in the establishment of early pulmonary inflammation with direct consequences on late evolution to fibrosis after lung injury. We demonstrate that IL-23p19, IL-17A and IL-17RA signaling are essential to pulmonary inflammation whereas IL-17F seems dispensable. Moreover we identified γδ T cells as the major source of early IL-17A, providing a new mechanism whereby IL-1 and IL-23 may mediate pulmonary fibrosis. IL-23p19 was identified for the first time as an essential mediator of pulmonary fibrosis. Importantly, IL-23p19 and IL-17A are upstream of TGF-β1 the central mediator of lung fibrosis. Our findings point on the potential role of IL-17-producing TCRγδ T cells in lung injury as a first line of defense that can orchestrate an inflammatory response leading to pulmonary fibrosis. Our study provides new information on the pathogenic role of IL-23 and IL-17 underlining the importance of targeting these cytokines in the development of new therapeutic approaches against lung fibrosis.

## Supporting Information

Methods S1 The methods for semi-quantitative PCR and flow cytometry.
(DOCX)
