# Supplementary material for: IL-1 and IL-23 Mediate Early IL-17A Production in Pulmonary Inflammation Leading to Late Fibrosis
Source: PLoS One. 2011 Aug 16;6(8):e23185. doi: 10.1371/journal.pone.0023185 (PMC3156735; doi:10.1371/journal.pone.0023185)
Supplement: Methods S1 — The methods for semi-quantitative PCR and flow cytometry. (DOCX) [file pone.0023185.s001.docx]

**Online supplemented material**

Semi-quantitative PCR

IL-17A, IL-17F, IL-23/p19 or IL-1β mRNA transcript levels from in lung of treated or control mice were assessed. Total RNA was isolated from less than 100mg of lung tissue previously snap-frozen in liquid nitrogen. RNAs were first extracted with RNA TRIzol reagent (Sigma) and further purified using a commercial kit (RNeasy, Qiagen,) following manufacturer’s instructions. 1µg of purified total RNA was used to generate first-strand cDNA synthesis by reverse transcription. These cDNA were subject to semi-quantitative PCR. An initial denaturation for 9 min at 95°C was followed by 30 (for IL-1β), 35 (for IL-23/p19, IL-17A and HPRT1) or 40 (for IL-17F) cycles of amplification. Amplification conditions were as follows: 30s of denaturation (94°C), 30s of annealing, and 30s of amplification (72°C). PCR products were resolved by electrophoresis on a 1.5% agarose gel. The electrophoresis gels were analyzed using a densitometric analyzer (ImageJ). Images were taken and band densities were measured and compared to housekeeping gene HPRT1.

Flow cytometry analysis

Lung mononuclear cells were isolated and stimulated for 4h with 10^−8^ M Phorbol Myristate Acetate (PMA, Sigma-Aldrich), 5.10^−6^ M ionomycin, and 20 μg/ml brefeldin A. Cells were incubated with CD1d-tetramer-APC (provided by NHI tetramer facilities), anti-NK1.1-PerCP-Cy5.5, anti-CD4-APCalexa750, anti-CD8-PB, anti-TCRγδ-APC, anti-TCRαβ-APC, anti-CD11bPerCp Cy5.5 (BD PharMingen) or isotype control antibodies. For intracellular staining, cells were fixed with 4% PFA, washed, and permeabilized with 0.5% saponin (Sigma-Aldrich) in PBS, then incubated with anti-IL-17A-PE, anti-IL-17F-PE, anti-GFP alexa488, anti-IL12p40-FITC anti-IL-23p19-alexa647 or isotype controls. The cells were washed, fixed and analyzed in a FACSCanto II (Becton Dickinson) by using FlowJo software.
